# Supplementary material for: The interactome of intact mitochondria by cross-linking mass spectrometry provides evidence for coexisting respiratory supercomplexes
Source: Mol Cell Proteomics. 2017 Dec 8;17(2):216–32. doi: 10.1074/mcp.RA117.000470 (PMC5795388; doi:10.1074/mcp.RA117.000470)
Supplement: Supplemental Data [file supp_RA117.000470_133922_0_supp_23978_fzffwf.zip › spectra_annotation/mito_spectra_annotation/156-2-1-1-6-1.pdf]

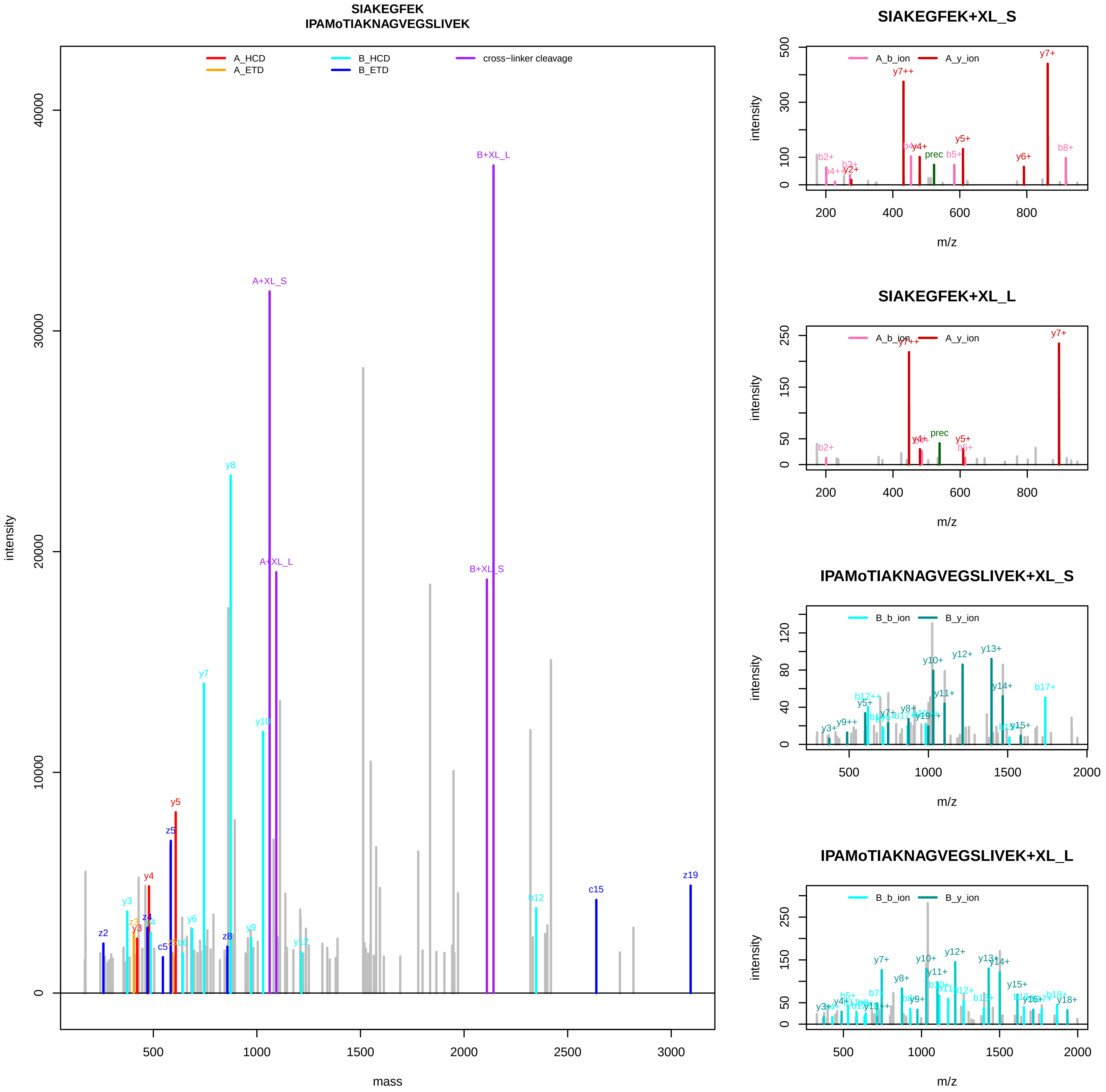

SIAKEGFEK
IPAMoTIAKNAGVEGSLIVEK
A_HCD
A_ETD
B_HCD
B_ETD
cross−linker cleavage
intensity
mass
0
10000
20000
30000
40000
500
1000
1500
2000
2500
3000
B+XL_L
A+XL_S
A+XL_L
B+XL_S
y8
y7
y10
y5
z5
y4
y3
z3
z4
z2
c5
y6
z8
y9
y12
b12
c15
z19
SIAKEGFEK+XL_S
SIAKEGFEK+XL_L
IPAMoTIAKNAGVEGSLIVEK+XL_S
IPAMoTIAKNAGVEGSLIVEK+XL_L
A_b_ion
A_y_ion
B_b_ion
B_y_ion
m/z
prec
y7+
y7++
y4+
y5+
y6+
y2+
b2+
b3+
b5+
b8+
y10+
y12+
y13+
y11+
y14+
b17+
y15+
y5+
y3+
y9++
y8+
y7+
y18+
y16+
